# Lynx: Automatic Elderly Behavior Prediction in Home Telecare

**DOI:** 10.1155/2015/201939

**Published:** 2015-12-09

**Authors:** Jose Manuel Lopez-Guede, Aitor Moreno-Fernandez-de-Leceta, Alexeiw Martinez-Garcia, Manuel Graña

**Affiliations:** ^1^Department of Systems Engineering and Automatic Control, University College of Engineering of Vitoria, Basque Country University (UPV/EHU), Nieves Cano 12, 01006 Vitoria, Spain; ^2^Computational Intelligence Group, Faculty of Informatics, Basque Country University (UPV/EHU), Paseo Manuel de Lardizabal 1, 20018 San Sebastian, Spain; ^3^Instituto Ibermática de Innovación, Sistemas Inteligentes de Control y Gestión Parque Tecnológico de Álava Leonardo Da Vinci, 9 – 2° Edificio E5, Miñano, 01510 Álava, Spain; ^4^Department of Computer Science and Artificial Intelligence, Faculty of Informatics, Basque Country University (UPV/EHU), Paseo Manuel de Lardizabal 1, 20018 San Sebastian, Spain; ^5^ENGINE Centre, Wrocław University of Technology, Wybrzeże Wyspiańskiego 27, 50-370 Wrocław, Poland

## Abstract

This paper introduces Lynx, an intelligent system for personal safety at home environments, oriented to elderly people living independently, which encompasses a decision support machine for automatic home risk prevention, tested in real-life environments to respond to real time situations. The automatic system described in this paper prevents such risks by an advanced analytic methods supported by an expert knowledge system. It is minimally intrusive, using plug-and-play sensors and machine learning algorithms to learn the elder's daily activity taking into account even his health records. If the system detects that something unusual happens (in a wide sense) or if something is wrong relative to the user's health habits or medical recommendations, it sends at real-time alarm to the family, care center, or medical agents, without human intervention. The system feeds on information from sensors deployed in the home and knowledge of subject physical activities, which can be collected by mobile applications and enriched by personalized health information from clinical reports encoded in the system. The system usability and reliability have been tested in real-life conditions, with an accuracy larger than 81%.

## 1. Introduction

Ambient Assisted Living (AAL) [1] is defined as the use of information and communication technology to shape intelligent living environments reacting to the needs of the inhabitants, providing relevant assistance, and helping them to live a fully independent life. End users are the stakeholders in the AAL ecosystem: citizens, formal and informal care givers, service providers, technology providers, and policy makers. The beneficiaries will be those people who wish to be able to avoid dependency on nursing homes, preferring to continue to live independently in their own homes. Assistance might be needed in any aspect of daily life, from health safety and security to social integration and mobility support. The steering board of the European Innovation Partnership on Active and Healthy Ageing (EIP-AHA) asserts [2]: “ICT solutions can prolong independent living of older people and extend the time they remain active and safe in their preferred environment. They also have a huge potential to enhance social inclusion and participation of older people, reduce depression rates, enhance quality of work for carers and make overall care provision economically sustainable (e.g., by avoiding and reducing hospital stays).”

Health knowledge about the elder's state is the starting point to identify behavior patterns and to assess his/her status like taking medication, activity recommendations, social interactions, early memory loss, disorientation, falls at home, and symptoms of weakness, tiredness, or fatigue. It can be stated that we are looking for an Intention Detection System. Various experiments on AAL environments have demonstrated the possibilities and the complexities of intention detection [3]. User action measurement and real-time monitoring of user experience enable a new range of innovative systems and key enhancements to existing products [4].

The main objectives of Lynx, the system described in this paper, are as follows:

- Develop a knowledge management system able to store and understand the user clinical status, activity, context, and situation aware, allowing to integrate this semantic information in the intelligent system, in order to detect health abnormal events.
- Create intelligent monitoring services of an elder and his medical issues, so that the system adapts to him, creating automatically rules that determine the usual values for each individual and evolve with the subject under monitoring, so that they are always up to date. These rules allow launching fully customized alerts without human intervention.
- Create a telecare third-party system based on an expert system and an inference engine that can automatically detect dangerous situations decreasing false positives, firing events only at abnormal circumstances.

The critical task of the system is the automatic profiling of user behaviors. Several systems were introduced in recent years to address elder-care issues, principally fall detection systems [5–9]. However, most of these systems either are too expensive for mass use or are of low quality. Most commercial solutions are capable only of fall detection [10]. Previous efforts have been done addressing this problem by our research group [11] based on ontologies [12], but the automatic data capture from electronic health records (written by medical personnel in natural language) and automatic medical summaries creation has not been considered until the present work.

The remainder of the paper is organized as follows.  Section 2 introduces the system architecture. Sections 3 and 4 give details about the system data-source types.  Section 5 describes the automatic data capture from health record in natural language for creation of medical summaries, while Sections 6, 7, and 8 explain the intelligence system. Up to this point, each section has explained each one of the different modules of the system.  Section 9 discusses the results of the real implantation of the system. Finally, Section 10 presents our conclusions and future work.

## 2. System Architecture

Context Awareness (CA) [13, 14] definitions focus on the situation of an entity, that is, the user's context, or the context of the devices carried by the user. Context includes location, identity, activity, and time. Recently the viewpoint has shifted towards a notion of the user being part of a process or ecology, as exemplified by Ambient Intelligence (AmI) [15]. In AmI vision devices interact to support people in carrying out their everyday life activities and tasks in a natural way on the basis of information and intelligence that is hidden in this network of interconnected devices. Research in smart environments is often related to AAL attempts to extract information about people well-being (sleeping, being awake, daily rhythm, falls, or level of general activity) from the various sensors, often including cameras [16].

The Lynx system is designed to fulfill two main requirements. First, the extraction, transformation, and load of sensor information are to be carried out in a simply way: the sensor is plugged on the network, and its raw data is automatically integrated in the platform. Second, the platform must be able to measure the elderly's habits in order to track his behavior to find deviations from their daily tasks (wake-up times, sleep habits, diary strolls, etc.) or regarding their health situation and provide a detailed summary to the caregivers and the family about their evolution.

**Figure 1:**
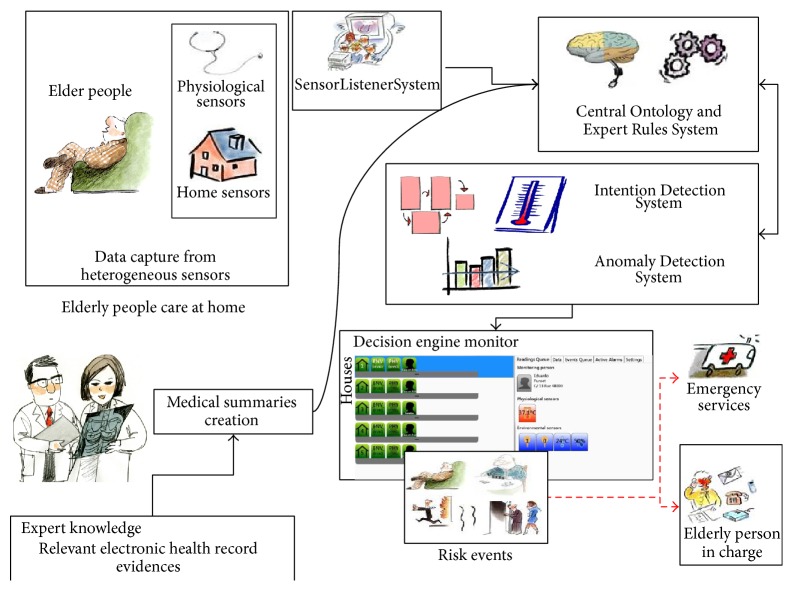
Lynx artificial intelligence platform over real housing with care systems.

Figure 1 shows how the system is organized in five main modules, which are discussed in the following sections:

- Data Capture from Heterogeneous Sensors.
- Relevant Electronic Health Record Evidences.
- Central Ontology and Expert Rules System.
- Intention Detection System.
- Anomaly Detection System.

The overall operation of the system represented in Figure 1 can be explained as follows: sensors collect and send information through the “SensorListenerSystem” when they are activated (by, e.g., presence, smoke, or opened doors) to the Central Ontology and Expert Rules System, which checks whether this information generates a status change over previous values. If this new information generates a status change over previous values, the event is recorded in the central database system. The collected information, depending on the type of the sensor, is checked with end user configured thresholds in a rule editor. If the taken measures do not meet those thresholds, a technical error event is generated by the system. Usually, since the sensors are very simple (the activation information is binary), this check is not usually necessary, only in physiological sensors. Finally, the correct data are transformed from raw data to context data (e.g., being at kitchen in the morning after a shower, it is translate to “taking breakfast”) by the Central Ontology System and its rules. This context stage is, finally, compared with the elderly usual behavior (based on the Intention Detection System and the Anomaly Detection System). If they do not match, the system will generate alert events, and, according to their pathologies (medical history), the Expert Rules System module provides concrete recommendations for action (notification to family, dialing to an ambulance, recommendations for improvement directly health habits, etc.)

## 3. Data Capture from Heterogeneous Sensors

The main component of this platform at the gateway level is the so-called Home Box Service (HSB), a structured software system, flexible and hardware platform independent, which is the system kernel processing the remote data received. Regarding the technologies involved, we use the UPnP [17] protocol which does not only cover internet protocols such as TCP/IP, HTTP, SOAP, UDP, or XML, but also integrate Zigbee, USB, IEEE802.11, BT, BLE Wi-Fi, and security considerations using security techniques like X.509 certificates. Moreover, this protocol is open and it can be extended (e.g., to define a specific type of message adding extra attributes). The Lynx system contains three types of sensors: environmental, physiological, and embedded service and audiovisual sensors, connected to one or more hubs.

Lynx has been admitted in universAAL [18], the European platform of reference in telecare systems. There is much more supplementary information about technical specifications of data capturing in previously published papers [19, 20].

## 4. Relevant Electronic Health Record Evidences

Caring for elderly requires a multidisciplinary approach and may include monitoring of the health status of elderly and the management of chronic conditions and the inclusion of one or more medications prescribed for regular use. This complex context must be taken into account in a telecare system [21]. Personal medical knowledge of elderly people in a teleassistance system allows improving the quality of their life habits, predicting dangerous situations derived from their pathologies. This knowledge, enriched with actual sensor information, is both manual and automatically stored in a specialized medical-telecare context-aware ontology representing information in a hierarchical way, with a dynamic and variable structure, which can be automatically updated “online” with new clinical information, new kinds of sensors, or new units of measurement. Also, this ontology implements the rules inferring new knowledge based on the raw data, health status, medical or behavior recommendations, and finally the expert system alerts rules.

Semantic networks implemented in ontologies allow for concept disambiguation [22], but even using huge medical resources collection of relations is not enough to create semantic networks. These relations are based on cooccurrences and do not contain any systematic description of the ontological concepts. Ontology itself may be used as a complex set of concepts joined in a parent-child structure, where each concept has been linked with some possible synonym, being most useful to resolve semantic conflicts of ambiguity in a concrete context. To be able to prioritize, we are using a rule-based system that has facility for explaining inferences as a framework for supporting clinicians at the point of care [23]. Given the large amount of information to be managed, we need to use storage techniques able to manage efficiently large volumes of data (data warehouse) and provide easy access and efficient management of the information (big data paradigm) [24]. So, in this project, we are using a no-SQL database, storing the information in a triple mode over the Virtuoso system [25, 26].

## 5. Automatic Data Capture from EHR Natural Language: Medical Summaries Creation

Nowadays, electronic health records (EHR) store most of the patient evolution information in natural language reports (80% of relevant information), written by the medical personnel, so that the percentage of structured information containing quantitative data is minimal. EHR systems can be combined with Diagnostic Support Systems (DSS) to allow order entry and provide alerts on errors of drug dosage, allergies, and contraindications, guided selection of diagnostic tests, reminders for procedures, tests, visits, computer-assisted diagnosis, and trend analysis. The extraction of the information is not an easy task and requires the development of correlation, noise reduction, and inference algorithms. The standardization of EHR is not a trivial problem. The coding of data within EHR and clinical databases is essential for sharing and exchanging across heterogeneous systems. Challenges to adoption of terminology standards [27] include the use of local coding vocabularies and idioms and efforts to map from disparate systems to a given standard, as well as identifying the appropriate standards for medications, laboratory, and other diagnostic tests and problems. In general, it involves the use of a combination of approaches (automated, semiautomated, and manual) and terminology systems (Master Drug Data Base (MDDB), RxNorm, Systematized Nomenclature of Medicine-Clinical Terms (SNOMED CT), and the Unified Medical Language System (UMLS) Metathesaurus) for identifying SNOMED CT concepts and hierarchies as well as UMLS Concept Unique Identifiers (CUI) for medications [28].

**Figure 2:**
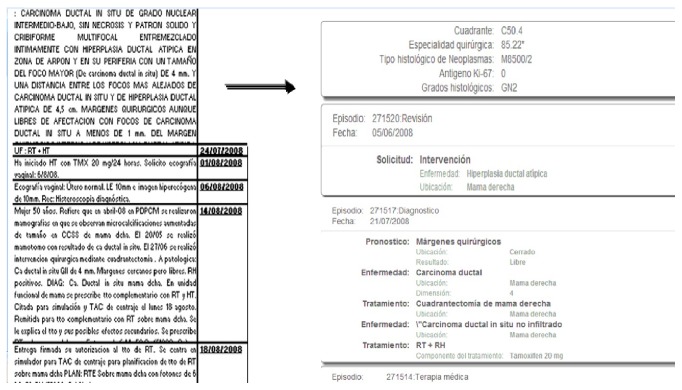
Data capture from clinical records.

It is not easy to identify the principal diagnosis or disease in natural language over EHR fields. There are descriptions about the family history, secondary diagnosis, and a constellation of comorbid diseases. We have developed a preclassifier to detect the main disease and diagnosis, the principal medical procedures, treatments, and medications impacting directly in the user's life, embedding it into the teleassistance platform as a new indicator. Normal age-related changes may be accompanied by chronic health problems such as diabetes or heart disease. Managing many such chronic conditions may include one or more medications prescribed for regular or daily use. Combined, these factors increase the complexity of telecare systems [29]. Usually, this information is in several clinical records stored in a not structured way but is very relevant in order to supply an optimal service. As seen in Figure 2, Lynx system is able to translate literal medical texts into a semantic structure system (the Central Ontology), in order to find the correlations between the clinical diseases, treatments, and drugs with the lifeday personal requirements of each elder.

There are mathematical techniques that are used to capture the semantic structure of documents based on correlations among textual elements within them [30]; however, inside Lynx system we propose a new method for creating medical summaries, supported by a statistical/semantic hybrid system. The annotation algorithms work over different sources, languages, and data types, based on vast text information sources (over the different natural medical language written clinical histories and medical reports comprising the broad set of medical disciplines and the different medical fields and tests). The achievement of this goal involves the following actions:

- To extract information from natural language medical reports in EHR that comprise a broad number of medical context and a great variability among the different medical fields, countries, and even hospitals.
- To define a new model that is able to comprise, normalize, and structure the information contained in the different clinical reports generating a unique structured descriptor in a standard format.
- To enrich the information from other sources, both external and internal, of heterogeneous data.
- To compress the generated models and descriptors to support compactness and real time response and to improve inference strength in semantic summaries.
- To develop a semantic ontology model that is able to structure, normalize, enrich, and compact the written clinical histories, test reports, and clinical records regardless of the medical discipline, country, language, hospital, and practitioner. This task will focus on the modeling of relevant information for clinical practice applications, for instance, the integration with other clinical collected data, and use this information for search new medical researches. Our key involvement focuses on linking to clinical IT and to the knowledge collected in clinical practice.

The system takes into account the correlation of all the concepts in the clinical records about the primary diagnosis of the patient, by calculating the different concepts vectors with the “tf/idf” frequency algorithm. Later, we run a “map” process which will analyze the direct correlation of secondaries variables with the principal concepts, extracting their relationships and recoding them under the Unified Medical Language System (UMLS) Metathesaurus. The UMLS Metathesaurus is a large, multipurpose, and multilingual vocabulary database that is organized by concepts. The current release comprises more than 1.5 million biomedical terms from over 100 sources. Synonymous terms are clustered together to form a unique concept or cluster. Concepts are linked to other concepts by means of various types of relationships, resulting in a rich graph. The Semantic Network provides a consistent categorization of all concepts represented in the UMLS Metathesaurus as well as information about the set of basic semantic types, or categories, which may be assigned to those concepts. Lynx Semantic Network contains 133 semantic types and 54 relationships.

Sharing common understanding of the structure of information among people or software agents is one of the most common objectives developing ontologies [31]. For example, if there is a set of different specialized medical databases (containing medical information), specialized genomic databases (providing genomic information), and other statistic databases sharing demographic and economical information regarding the population in a certain situation, there could be services able to share and publish all this information under the same ontology and vocabulary. So, at the end, computer agents could extract, join, aggregate, and infer new information from these different sites. The agents could use this aggregated information to answer user complex and cross-domain queries or as input data to other applications. Enabling reuse of domain knowledge was one of the driving forces behind recent surge in ontology research [12].

**Figure 3:**
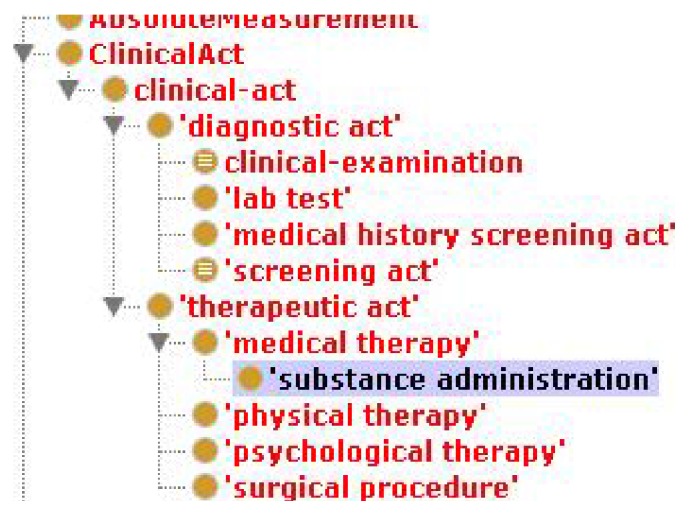
Medical observations in the summaries ontology.

First, we built a large general medical ontology (illustrated in Figure 3) merging ontologies from oncological, a heart failure, and a general medicine contexts, all based on OpenEHR archetypes, used as the main basis to build the semantic classes, subclasses, and properties. OpenEHR archetypes are reusable definitions for fragments of clinical information. Translating them to ontology languages enables the use of inference and mapping to existing formal ontologies in the biomedical and clinical domains. Once these clinical archetypes are translated into Ontology Web Language (OWL) objects, we combine them to set up our Summaries Ontology which becomes the starting point of the inference process. It is shown that the OWL technology (a semantic storage format) achieves high efficiency, accuracy, scalability, and effectivity [32]. Also it is necessary to store the patients evolution (clinical actuations) named “observations” in OpenEHR. To join these concepts into our ontology, we enrich our base ontology with a specific and a universal “observation” ontology, including identifying information model elements, vocabulary concepts, and data types from key standards such as HL7/RIM, Detailed Clinical Models (DCM), Clinical Document Architecture (CDA), and the Study Data Tabulation Model [33].

Figure 3 shows that the ontology is able to store information in a semantic format, episode by episode, about what the most important concepts extracted from each episode are. Examples of such extracted concepts are whether there has been a secondary diagnosis, the episode speaks about a previous general diagnosis, there has been any kind of treatment, medical, or surgery procedure, he has recommended a drug in particular, the treatment has been modified, any specific pathology has been detected, or simply the patient has been informed of something. Also, each episode has a type of “Clinical Action,” as “diagnostic act,” “therapeutic act,” and so forth, extracted from the OpenEHR standard ontology. The Lynx ontology supports the CEN/ISO EN13606 norm, that is, a European norm from the European Committee for Standardization (CEN) designed to achieve semantic interoperability in the electronic health record communication.

The general algorithm to create summaries has the following steps:

1. Perform the text preprocessing steps: stemming, stop-list, and spell-checking, either correcting or removing strings that are not recognized.
2. Use MetaMap [34] with very restrictive settings to avoid highly ambiguous results when mapping text to UMLS ontology and try to expand some acronyms.
3. Use UMLS relations to create first-order classes, adding only those types of relations that lead to improvement of classification results.
4. Annotate the concepts on the summaries ontology with a set of rules joining automatically pair concepts with their relationships or properties.

Table 1 shows through an example the result of the described algorithm. It can be seen that the electronic health record written in natural language which is the original source of information, and the diagnostic and the treatment derived from it expressed in UMLS.

The use of a No-SQL database to store Resource Description Framework (RDF) and Web Ontology Language (OWL) descriptions makes it possible to query, manipulate, and reason about the data with standard tools, such as OWL reasoners and languages (e.g., the SPARQL Query Language for RDF).

### 5.1. Text Preprocessing Steps, MetMap Annotations, and Concepts Weights

**Figure 4:**
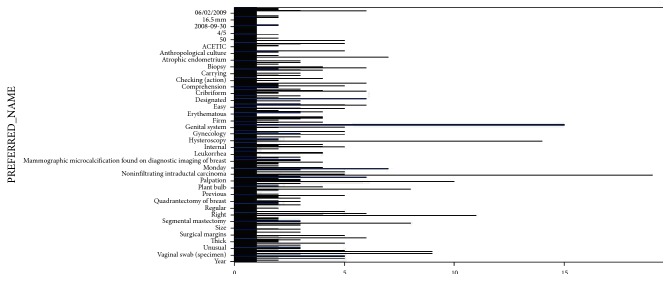
Absolute frequency of terms found in clinical records.

**Figure 5:**
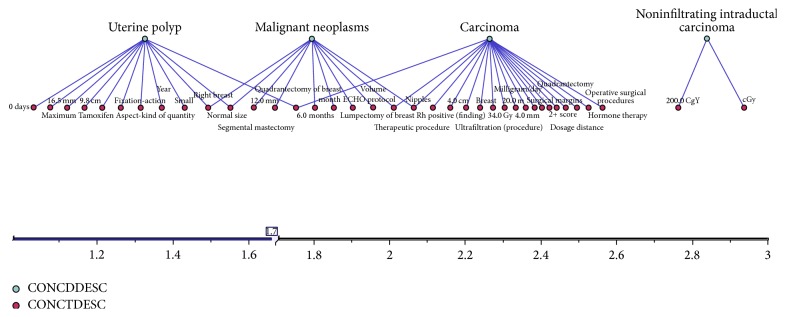
Grouping by principal diseases.

The EHR typically contain description of different episodes written in natural language by the medical staff; often they are multilingual (English, Spanish, etc.) with their diagnostic impressions, treatments, procedures, and so forth [35]. The first step is to determine the break points in each episode regarding a principal disease, a principal treatment, or the medical recommendations and the transformation of the concepts found in every group over UMLS codes in an indexed process according to the following steps:

- For indexing, there are public tables with “stop-words” (not relevant words) and some dictionaries to help in the automatic translation between concepts (one or more words regarding a medical disease, procedure, treatment, drug or observation) and codes, scripts errors, abbreviations, or names of very local concepts (e.g., Rt is radiotherapy). However, these codes are plain; that is, their importance is based on the frequency, assigned to a group, and basically, it is proportional to the way of occurrence of the terms in each document or group and inversely proportional to the appearance of such terms where they have the whole information set.  Figure 4 shows an example of the absolute frequency of apparition of different terms in a set of clinical records. To incorporate semantics [36] that can “understand” what really means the medical staff, two external sources are used: hierarchies of UMLS coding and a medical dictionary of synonyms and acronyms. Not all episodes of a medical record have the same weight within a patient history, and also within each episode, not all sentences are relevant: there may be references to background, not medically significant observations or not sufficiently relevant to appear in a medical summary, although subsequent to an analytical process. Therefore, the system records all the concepts in the ontology, but later, in the presentation of the final summary, only the information of those that are the most relevant episodes is shown, that is, those that have objective correlation with main diagnosis of each patient. The whole text in the clinical record is split into sentences using the tokenizer, which carries out such transformation following the grammar of the natural language. The tokenizer is part of speech tagger and sentence splitter modules of the General Architecture for Text Engineering (GATE) architecture for text engineering and transforming into the UMLS codification.
- Each UMLS concept belongs to a hierarchical class, that is, the key of the Lynx system. There is a statistical model that calculates the weight of the episodes analyzing the “td/idf” frequency of each concept into an episode, taking into account the weight of the class to which each concept belongs. A diagnosis does not have the same weight compared to a treatment, a part of the body, a drug or compared to different combinations of different concepts. With our weighting algorithm the Lynx system selects all entries and chooses the most relevant episodes, and, within them, it selects the main and secondary diseases for each episode (see Figure 5). In addition, the relevant knowledge is not in the amount of concepts in a basic list with their weights but how these concepts are related between them. Each pair of concepts is transformed into a relationship join (a graph), and a graph corresponds to a different category of information (e.g., diseases, symptoms and signs, or medicaments). In order to create this graph, we need to score in the ontology concepts in “triplets” according to a set of high level rules defined in the inference system. In this way, the system is able to transform the plain database concepts in a hierarchical semantic structure, from medical annotation engine developed in the Lynx system.

So, the annotation process is as follows:

- Extract relevant tokens from sentences along different episodes in the whole health record (stop-words, dictionaries, translates, UMLS codes, etc.).
- Assign the UMLS concepts to their principal classes. This research requires a proper mechanism to select them in order to support further tasks like adding semantic rules by means of Semantic Web Rule Language (SWRL) and launching inference, setting bindings to terminologies, validating archetypes, and so forth. Thus, it should be noted that describing constraints as human-readable comments should be avoided as far as possible given that they cannot be used by semantic reasoners.
- Calculate the weight of each episode of the health records and the weight of each concept into the episode.
- Calculate, from each set of sentences, when the text is talking about the same diagnosis, treatment, or procedure and grouping sentences by principal or secondary diseases.

With this plain information, we are able to transform the concepts into a hierarchical (semantic) format, in order to

- annotate only the relevant information,
- present to the medical staff only the relevant relationships between concepts,
- analyze unknown relationships between diseases, procedures, treatments, and personal patient data as familiar issues, gender, age, demographic or economical situations, personal context, drugs, and so forth,
- detect unknown relationships between different episodes along the time; the statistical models, mainly on clinic records, lose knowledge if they do not take into account the evolution over time. Time evolution is a variable which has not been deeply studied within repetitive sets of data in the medical domain. the Lynx system uses machine learning with sliding windows to solve this issue [37].

### 5.2. UMLS Relations and Filling the Ontology

**Figure 6:**
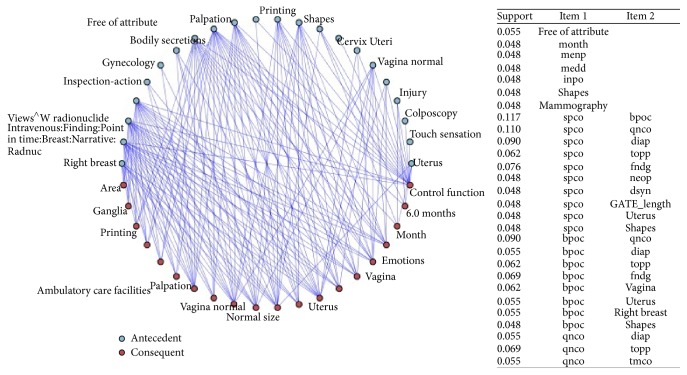
Minimal relevant gain between principal UMLS classes.

Once the concepts are grouped by primary and secondary diseases, treatments, or procedures and extracted from a relational database, some semantic rules are executed to convert in triplets (sets of subject-predicate-object) the relationships found in the different principal groups. In addition, each triplet is assigned to a principal disease, if it exits in the group, or to a secondary disease, depending on its content. Each concept in the clinical record belongs to a UMLS class (see examples of hierarchies in the table of Figure 6). Previously, statistical learning processes the entire corpus of EHR has been carried out. With a weighting and association algorithm we selected those classes or pairs of semantic types or related hierarchies with a minimal relevant gain to be taken into account in the general summaries, as shown in Figure 6.

**Figure 7:**
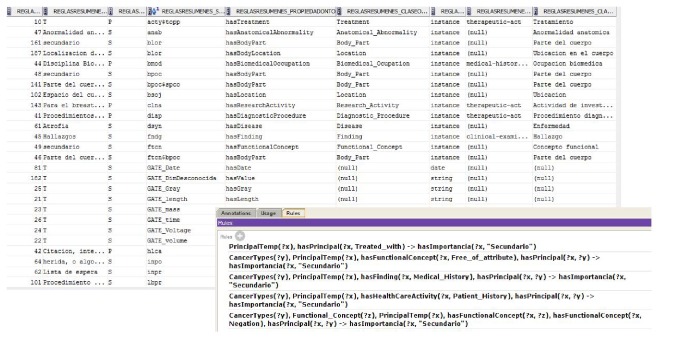
Annotation rule system.

Each unique class or pair of semantic types is manually assigned to an annotation rule.  Figure 7 shows this assignation. For example, the first line means that when in the same sentence there is a type of concept named “acty” (which means “activity”) and close to it there is another concept of type “topp” (which in turns means “therapeutic procedure”), then the real instance in the ontology is that “patient” “has treatment” “topp” (where “topp” is instanced by means of the therapeutic procedure found in the text).

So the system is able to recognize the semantic types and to infer new semantic instances that are the real annotations in the ontology. The system uses a reasoner engine (Pellet), so once all the semantic instances relating to the same group of diagnosis, treatment, or procedures are recorded in a temporal memory, the system runs several rules to determine if the triplet within that group belongs to an earlier principal disease, that is, a new main diagnosis or a secondary diagnosis. Finally, all the triples processed are stored in the no-SQL structure and can be used directly for consultations or queries by medical practitioners, almost in a natural language format in the style of queries that can be similar and linked to other platforms like “LinkedLifeData” [38], based on semantic “endpoints” to access the information. There is also a web platform where doctors can analyze and summarize medical history based on the dates of diagnoses and main treatments, so that medical staff can see the evolution of all patients in a graphic format and, secondly, see at a glance the diagnostic, procedures, treatments and relevant abnormalities of a patient in a digital format.

Reasons for sharing and reusing semantic (SWRL) rules include the ones listed below:

- Interoperable decision support: the ability of systems to reliably communicate with each other regarding clinical decision support, to encourage the development of interoperable mechanisms for triggering critical aids to decision making like alerts, reminders and monitoring tasks that improve effectiveness and reduce clinical risks.
- Inheritable compatibility: given the archetypes' capability of being defined as specialization of more general archetypes, a SWRL rule originally designed according to the OWL version of a parent archetype is also applicable to derived archetypes.
- Fostering semantics for clinical guidelines: the introduction of SWRL rules and inferential mechanisms together with the archetypes expands the boundaries of the declarative knowledge that can be migrated from clinical guidelines to healthcare information systems. In this way, a means for standardized representation, reuse, and execution of the essential fragments of declarative knowledge contained in clinical guidelines is provided.
- Specialists' empowerment: to enable domain rules and guidelines to be modeled in a formal way by domain experts. By defining the declarative knowledge they work with, they can gain direct control over their information systems.
- Consistency checking: rules integration can offer consistency checks to help guaranteeing data correctness in EHR fragments.
- Archetype validation: to support archetype validation and inconsistent restrictions detection.
- Full semantic interoperability: for all abovementioned reasons, integrating rules with clinical archetypes and EHR is an essential step towards extracting relevant information into summaries.

**Figure 8:**
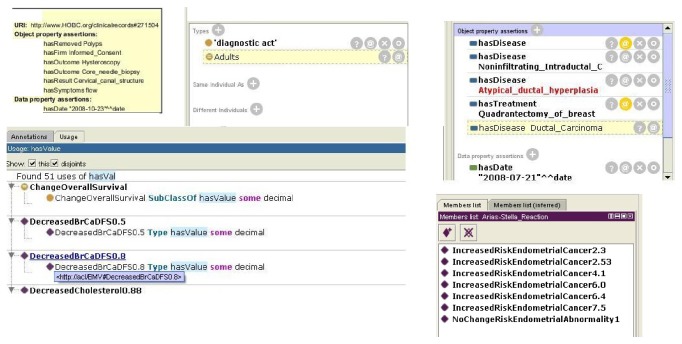
Clinical record in a semantic store (semantic tool view).

The advantage of the system is that the rules for concept weight calculation and for creation of relationships between concepts, joined to the rules that determine whether a disease, treatment, or procedure is the principal or not in the context of the patient, are fully configurable by the medical staff, without computer, statistical, or semantic knowledge, therefore, in a very dynamic, elastic way, allowing a variation of different medical contexts.  Figure 8 shows the semantic tool view of a clinical record in a semantic store, instantiating the ontology of Figure 3 with annotated values of a concrete patient. More specifically, Figure 8 shows how is instantiated a case of medical diagnostic (“diagnostic act”) through an “Atypical_ductal_hyperplasia” diagnostic in an adult with a “Quadritectomy_of_breast” treatment, and this medical diagnostic corresponds to a “clinical-act” in the general ontology of Figure 3.

### 5.3. Joining the Summaries and Teleassistance Central Ontology

**Figure 9:**
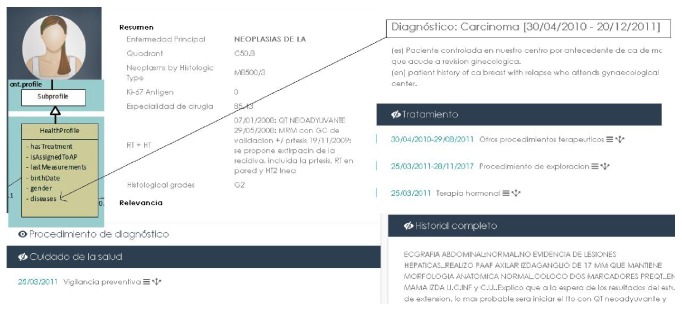
Joining summaries and central ontologies.

The ultimate goal of automatic summaries is to feed the telecare platform with the most relevant clinical data obtained by an unassisted way from medical summaries and move the therapeutics procedures, treatments, or medical recommendations from medical summaries to universAAL ontology. So, the anomaly engine and the predicting intentions engine are capable of learning about personal living habits of the patients, highly correlated with their clinical conditions and prescriptions. To build this integration, thanks to semantic annotation process, we need only to join the most relevant concepts (principal diseases, diagnosis, treatment, and procedures) in the control platform (see Figure 9) with a “same_as” link between ontologies, by the paradigm of Linked Data recommendations [39].

## 6. Central Ontology and Expert Rules System

Expert systems represent formal knowledge to solve human problems. This type of systems is applicable to any domain and is present today in nearly any application that requires high computational cost to automatize processes with some reasoning. Expert systems are suited to specific tasks which require a lot of knowledge derived from a particular domain experience as diagnostics, instructions, predictions, or advice to real situations that arise and can also serve as training tools, mimicking the human behavior.

By their nature, the Semantic (Knowledge-Based) Management Decision Support Systems (MDSS) work over structured data representation (schema). The knowledge is persistent in data-storages, and the expert knowledge (system rules) is heuristic evidence based rules, with reasoning capacity using an inference engine. This means that the rules are well-known and always are true (an uncertainty factor in the MDSS systems by their definition is not possible, in principle). The use of the Resource Description Framework (RDF) standard (and thus its associated representation machinery such as RDF schema and OWL) offers the possibility of making inferences when retrieving and querying information, in a way very similar to human natural language, being the advantage in query-answer systems. Although OWL automated reasoning does not scale up for use in large knowledge bases, researchers and practitioners have just begun to explore the problems and technical solutions which must be addressed in order to build a distributed system. On the other hand, there are nonknowledge-based MDSS, which learn from raw data (semi/unstructured) and are based on probabilistic techniques: patterns are taken as examples or cases in the past and the system has learning and probabilistic prediction capability. The direction of the last researches [40] is merging both engine capacities in a hybrid motor platform.

**Figure 10:**
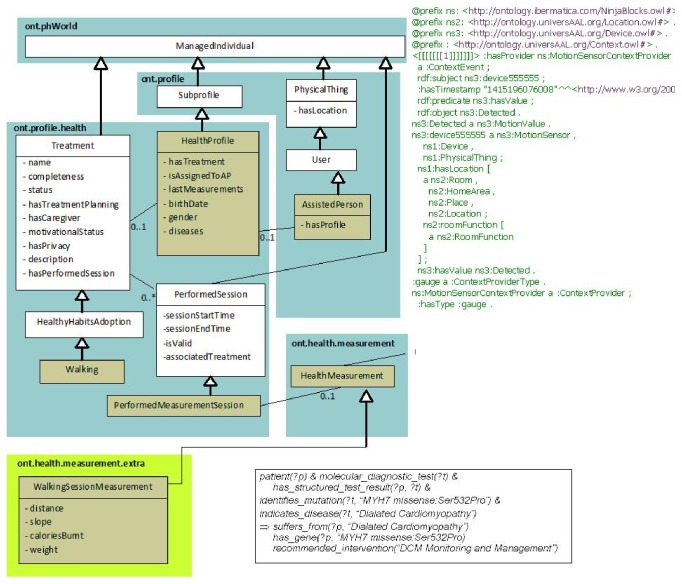
Ontologies and rules in the expert system.

“Semantic smoothing for language modeling” emerged recently as an important technique to improve probability estimations using document collections or ontologies, and this was the way followed to design this system. This is the technical way in this project. In Figure 10 we show the developed two joined ontologies, that is, the “Home Care” ontology (right part) and the “Health Habits Recommendation” ontology (left part). The sensor and the clinical records system fill them automatically on real time processes, based on a set of semantic rules. Therefore, the Expert Rules System has two main goals:

1. Fill the ontology with the sensor and clinical records raw data by means of “process rules.” Since these rules are easy to adapt, alter, and maintain, this feature makes them an attractive solution for nonexpert caregivers. The caregiver is able to directly define and modify the rules that specify the behavior of a system in a given situation. For example, context-aware behaviors could be specified by a rich set of rules. In addition, the use of rules on top of ontologies can enable adaptive functionality that is both transparent and controllable for users [41]. Some examples of rules which are used to fill the “Health Habits Recommendation” ontology are the following:
  - Transformation of raw data into complex concepts, for example, “at noon in the kitchen over half an hour, the patient is eating.”
  - Avoid data processing outside certain limits or thresholds set by the caregiver.
  - Simple warning rules: more than 2 hours in the bathroom throws an alert.
  - Medical inferences: “if cholesterol is more than a threshold, the patient is suffering from hypercholesterolemia.”
2. Execute expert rules (see Figure 10, right bottom part) in order to suggest health recommendations to the elders or send alerts about abnormal situations about the elder's behaviors, by means of a set of “home care rules.” Context-aware applications should continuously monitor the elder's environment in order to detect changes and react to them. So, rule-based architectures offer flexibility to tackle the variability of the environment and support the reconfiguration of systems according to changing needs without requiring reprogramming nor human intervention. Rule-based approaches are suitable for highly dynamic context-aware services [42].

Both set of rules are running over the ontologies with a semantic reasoner (Pellet), and the main advantage of the system is that it is open to future development: the functions are out of the kernel code, and the program flow or the alarms system can be modified changing the rules; that is, it is not necessary to change the code of the system kernel. So, this can be done by an expert who does not necessarily have to be a programmer. Defining personalized and adaptive elder interaction/behavior models is a key challenge when considering the issue of analyzing or predicting elder intentions. The intention aware elderly care application can predict the activities of the elder based on historic usual activity, profiting automatically the elder usual activity information. So, the system has a hybrid “inference engine” that joins heuristics rules (first conditions to check without elder data) mainly with a supervised system, in order to discover new rules, adapting the heuristic rules to the personal behavior changing, with an unsupervised system, with two objectives:

- Detect unknown alerts (not registered in heuristics nor supervised system).
- Join similar users' behaviors in homogeneous groups (segment usual activity, customizing automatically the user usual activity, state, or profile), in order to send new extracted knowledge to the medical and care agents about the general behavior of their patients.

In general, after the home installation, the inference machine runs the following tasks:

(i) Step 1: identification of the observable behaviors of the user.
(ii) Step 2: matching the heuristics rules to detect abnormal facts.
(iii) Step 2: start the learning process during the learning period.
(iv) Step 3: put the learnt behavior model into practice by applying the described process, modifying the heuristic general rules by heuristic personal rules.
(v) Finally, back to Step 1.

Human actions are influenced by context, knowledge, or experience of dependencies between actions and by expectations of how the situation is going to develop [43]. For example, if it is raining, even though it is summer, the elder probably does not go outdoor unless he likes walking in the rain. These subjective observations should be treated and the inference system must be able to learn these behaviors. In fact, the inference engine is an “Activity Tracker” system, and it is intended to track daily activities of the elderly via automatic or manual inputs. The real system uses some cases running over the “Activity Tracker” for example:

- The system infers whether the elderly is eating at a specific time of day. The indoor localization system is aware of the time of day and the amount of time that the elderly stays in the kitchen. Electrical appliance usage is also taken into account to infer if the elderly has been preparing his/her meal. Contact doors on kitchen furniture is also used to track this situation.
- During the day, the system infers whether the elderly is doing the housework. The indoor localization system is aware of the time of day and the amount of time that the elderly spends tracking his/her position. Vacuum cleaning can also be detected.
- Physical exercise like hiking or strolling can be detected through the GPS-enabled smart-phone of the elderly. The position, velocity, and path will be analyzed to measure the amount of kilometers of each day.

The “Activity Tracker” system provides three services for the elderly activities intention aware API:

- Current activity: it is the activity that the elderly is performing at the present time.
- Last 24 hour activities: they are the activities performed during the last 24 hours split into 15-minute time slots.
- Next 15-minute forecast activity: the next activity that will perform the elderly based on the historic data of the elderly intention is inferred. This horizon was determined in a heuristic way, since 15 minutes is much more than enough for the purpose of the system, and the higher the horizon, the lower the accuracy (in this and in all prediction systems).

This service provides the likelihood of the tracked activities to happen and takes the activity whose probability of happening is higher than the other ones.

## 7. Intention Detection System

The main task of the Intention Detection System is the development of decision trees that are running on central server and, based on information collected by different sensors deployed in the home, make decisions about the status of the patients, depending on their health situation (extracted from clinical records), treatment procedures, level of stress, behaviors, risk of exposure to harsh circumstances, and so forth. To do this, as exposed in Section 6, we have developed an expert system which reflects the knowledge, about which is the best guideline of action as would be advised by a specialist in the area.

As each home user could adapt differently to his environment and could have a recent history of actions that involve a danger due to accumulation of negative elements that can affect him, it is important that the general rules generated by experts adapt automatically to each user inside the platform with implicit knowledge, so the system will personalize the information to each patient, their current parameters, and their last actions, allowing elimination of the false positives. These supervised models exploit the reasoning power of expert system to derive new knowledge and facts. The Intention Detection System reasons over the base knowledge to infer new facts, resolves context conflicts, and maintains the consistence. The situation of a user is derived from his personal context, but the context is derived from the aggregation of all the user's situations plus the environment situation, too.

Through these techniques of intelligent information processing, we are given special emphasis on the detection and prediction of anomalies (trend analysis, deviations in the data, etc.), such as lifestyle changes and poorly executed exercises.

One easy way to detect changes and behavior anomalies is to compare the actual situation or state of the elderly with one prediction of his state. In this paper, is proposed a 24-hour sliding window that is analyzed with a decision tree (a supervised machine learning algorithm), in order to predict the next user action over the time. For each time window, the decision tree takes into account a multivariate set of values generating a predictive model (decision tree) and extracts the next status of the user, with confidence and probability levels.

**Figure 11:**
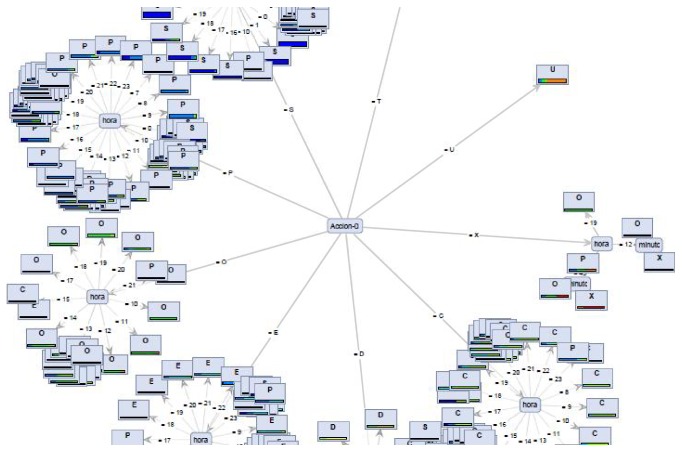
Personal behavior model per user.

Compared to the current technology for evaluating context-aware systems, we focus in particular on the quantitative evaluation of each one of the rules of our rule-based system with a temporal data set. The challenge of this proposal is a novel and distinctive base technology repository that has been developed in the treatment of time series and another algorithm repository for rule generation based on probabilistic rules directly from RDF semantic systems, without human knowledge, and automatic insertion of these rules in the central ontology again, assigning weights to the semantic confidence (triple-stores), in order to customize the personal behaviors of each user. The described system is proposed as a new development and addressing mode of the current telecare systems taking into account the characteristics and preferences of each person in such a way that a personal behavior is built. For example, Figure 11 shows the personal behavior model of a concrete elder. It is a decision tree, in which the main branch is the current action (Acción-0) carried out by the elder, being in the most of cases the hour (hora), the second condition to check in order to determine the future action (codified by a letter as explained in Section 9).

## 8. Anomaly Detection System

Usually, interest driven analysis tends to overlook unexpected patterns in data. To avoid this inconvenience the system contains unsupervised algorithms (clustering and association models). Data Mining deals with applications such as Anomaly Detection to prevent excessive consumption, pollution, escapes, and, in general, abnormal patterns of what an expected profile for each segment is.

To detect anomalies, we use the Local Outlier Factor (LOF) algorithm [44]. The algorithm compares the density of data instances around a given instance A with the density around A's neighbors. If the former is low compared to the latter, it means that A is relatively isolated; that is, it is an outlier. Such outliers are considered anomalous. With the aim of identifying and grouping those outliers, they can be classified by means of techniques based on statistical models or region density distances [45]:

- Statistical models: they are based on the field of statistics, given the premise of knowing the distribution of data. Based primarily on measurements of distances between objects, the greater distance of the object relative to others is considered an anomaly.
- Region density distances: based on the estimation of density of objects, the objects located in regions of low density and relatively distant from their neighbors are considered anomalous. The main feature is that generally it is considered unsupervised learning and a score is assigned to each instance that reflects the degree to which the instance is anomalous.

One of the tools necessary for the Anomaly Detection is clustering, which is to group a set of data, without predefined classes based on the similarity of the values of the attributes of different data. This grouping, unlike classification, is performed in an unsupervised manner, since it is not known beforehand of classes of the training data set.

**Figure 12:**
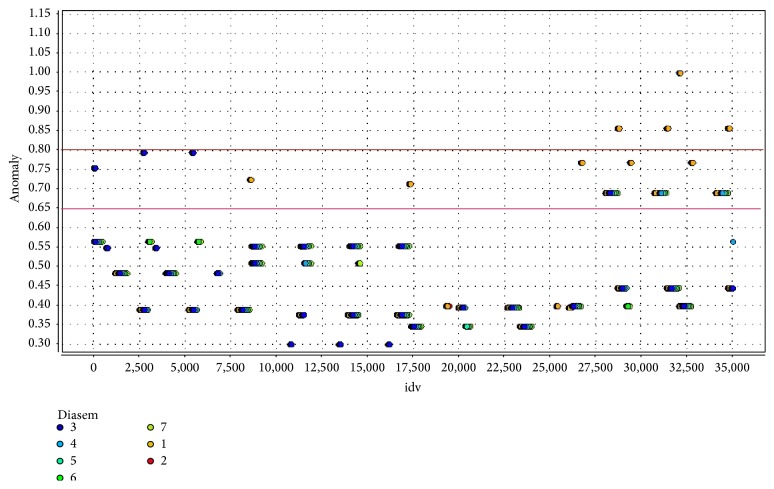
Anomaly Detection System.

The algorithm used to carry out the segmentation process takes as an input the data set (sensors data and clinical history) and the cluster model that was generated by a clustering algorithm (*K*-means). It categorizes the clusters into small and large clusters and the anomaly score is then calculated based on the size of the cluster the point belongs to as well as the distance to the nearest large cluster centroid. With this model, we can check automatically which are the* anomaly* events in the normal life of one person and detect which is the outlier event and why. This information is sent to the Expert Rule System to decide or not to throw one* alert* to family, caregivers, or medical centers.  Figure 12 shows the abnormal events of one user along one year, discretized into 15-minute periods. It can be easily seen that the anomalous behaviors usually happen in Sunday (orange dots): in this case the action which generates the anomaly is “going out of home” (coded as “O,” see Section 9), action that is not usual over the rest of the week. There are two red lines bounding the regions in which the* anomalies* are analyzed by the rule system to determine whether they are converted into* alerts* (the lower red line indicates a “low level” anomaly, while the higher one indicates a “high level” anormality). The expert rule system configured by the caregivers plays an important role because it determines that it should be considered as an* alert* or not (i.e., going out of home on Sunday could be discarded as an* alert*).

## 9. Deployment Results

Since the beginning of 2015, 60 homes have installed the system, checking online the users daily living over three different customer: dependent elderly people, elderly whose habits are worsening due to aging, and elderly people who are suffering the first symptoms of dementia. As previously exposed, the expert rules are running since the moment of the installation, but after a month capturing raw data, the system begins to obtain the behavior of the elderly. The steps through which the system gets their behavior are the following:

- At a first stage, the sensor raw data are processed by the expert system to determine which is the elder's context at every time, formatting the data into a structured table with the information about person, date, hour, and the stage in that moment regarding the user: Sleeping (S), Cooking (C), Eating (E), Doing Housework (D), Outdoor (O), Outdoor Sport (U), Using Tablet (T), Using Mobile (M), or Spare Time (P).
- At the same time, the system checks the physiological status of the users, as temperature, heart pulse, blood pressure, and so forth. In this way the data will be managed more compactly and only the relevant information to the alerts is managed (e.g., “the elderly has a fever unusual at 08:02 more than 15 minutes”). Initially the thresholds that are in the table are defined manually by the doctors (e.g., a temperature of 36,5°C), leaving the detection of statistical thresholds for later, where the average temperature threshold is modified by historical and statistical processes and unsupervised algorithms. These thresholds are able to modify the medical rules directly regarding the user personal historical set.
- The system also checks environmental sensors, such as smoke, temperature, and humidity, throwing alerts when activated.
- At every moment, the system is taking external data to integrate them into users data, with a number of values: Haze (C), Fog (N), Low Fog (N), Fog (I), Precipitation (P), Drizzle (L), Rain (U), Torn Rain (V), Tornado Sight (R), Rain Shower (H), Rain (E), Snow (E), Shower Hail (T), and Freezing Rain (T).

The first conclusion, as expected, is that the outdoor weather conditions are the most relevant in order to predict which will be the user behavior, in second correlation place (by a Chi Squared statistical test), after the hour of the stage, but before other indicators such as the day week, or even the month of action. With the temporal sliding window method, including the last action in order to predict the new stage of the user, the accuracy trust by the system using cross-validation is about 81,80%, as shown in Table 2.

Thus, it is shown that external data on local weather and data from past actions are representative to make predictions about the future status of the elderly immediately. Thus, if the prediction state does not match with the actual state and this situation has a significant score, the system sends an alert to remote care services to immediately launch protocols. On the other hand, the anomaly clustering method used is a complement to the supervised system, in order to detect not so usual behaviors.

## 10. Conclusions and Future Work

In this paper, we have introduced a behavior prediction system to be used in an home telecare platform. Such platform is an assisted living system for monitoring elderly people at their homes, with the final aim of providing a robust, easily deployable, and cost-contained solution to ensure the safety of the elderly. It obtains both physiological and environmental data through a multisensor infrastructure, connecting the home with both the carer and the family, being aware of the state of the elderly.

Our next steps in the closed future are two: on one hand, to transform this validated system into a reference product in the market of home telecare platforms and, on the other hand, reinforce the intelligent systems for analysis of anomalies and behaviors taking advantage of the health information to detect how the clinical records, medical diagnoses, and treatments are affecting the usual behavior of different patient profiles in their daily lives, specially, at home, and if these treatments are appropriate or not.

Finally, as a medium term step, we are researching on the challenge of creating a new method for automatic big data analysis over the medical summaries (obtained from clinical records, usually written in plain text natural language), in order to discover new relationships between diseases, medical procedures, treatments, and their correlation with home personalized medicine.

## Figures and Tables

**(a):** 

D	4755	4270	C0007124	1	dcis	neop	Noninfiltrating intraductal carcinoma

D	4755	4279	C0337354	1	Quadrantectomy	topp	Quadrantectomy of breast

D	4755	4280	C0222600	1	Right breast	bpoc	Right breast

D	4756	4298	C1176475	1	Ductal carcinoma	neop	Ductal carcinoma

D	4756	4299	C1318216	1	4	qnco	4

D	4756	4300	C0237753	1	4 mm	GATE_length	4.0 mm

**(b):** 

D	4757	4303	C0007097	1	Carcinoma	neop	Carcinoma

D	4757	4308	C0450367	1	4.5	qnco	04-may

D	4757	4309	C0237753	1	4.5 cm	GATE_length	4.5 cm

T	4757	4311	C0543467	1	Surgical	diap, topp	Operative surgical procedures

**Table 2:** Confusion matrix with previous stages.

Accuracy: 81.80%										
	True S	True P	True E	True T	True O	True C	True D	True U	True X	Class precision
Pred. S	7404	270	9	2	74	23	0	0	3	95.11%
Pred. P	313	2318	136	51	66	207	23	2	0	74.39%
Pred. E	16	175	851	84	6	68	124	0	0	64.27%
Pred. T	0	73	17	994	126	27	6	38	1	77.54%
Pred. O	0	87	44	6	843	119	1	72	14	71.08%
Pred. C	36	197	244	42	3	1321	56	0	0	69.56%
Pred. D	2	25	0	83	8	75	345	0	0	64.13%
Pred. U	0	28	2	0	59	33	2	242	5	65.23%
Pred. X	0	0	0	0	1	0	0	3	2	33.33%

Class recall	95.28%	73.05%	65.31%	78.76%	71.08%	70.53%	61.94%	67.79%	8.00%	
